# Physical activity and social anxiety among rural-origin university students in Southwest China: exploring potential associative patterns of body image satisfaction and social capital

**DOI:** 10.3389/fpsyg.2026.1879490

**Published:** 2026-08-14

**Authors:** Xiangbai Zhang, Weining Yang, Qi Xue

**Affiliations:** 1 Institute of Physical Education, Kunming University of Science and Technology, Kunming, China; 2 College of Arts, Yunnan Normal University, Kunming, China

**Keywords:** body image satisfaction, physical activity, rural-origin university students, social anxiety, social capital

## Abstract

**Background:**

Rural-origin university students in China face distinct psychosocial challenges, with social anxiety being particularly prevalent. Although physical activity is consistently associated with reduced social anxiety, its underlying mechanisms in this population remain unclear, especially regarding the potential statistical mediating roles of body image satisfaction and social capital.

**Methods:**

A cross-sectional survey of 965 rural-origin undergraduates was conducted in Yunnan Province, China. Validated instruments assessed physical activity, body image satisfaction, social capital, and social anxiety. Correlation, regression, and associative mediation analyses tested direct and indirect associations.

**Results:**

Physical activity showed a direct negative association with social anxiety (55.5% of total effect). Three indirect pathways emerged: via body image satisfaction (16.5%), via social capital (18.0%), and a sequential pathway (physical activity → body image satisfaction → social capital → social anxiety; 10.5%). The overall association was modest (unstandardized coefficient = −0.016 for item-average scores), suggesting limited standalone clinical utility.

**Conclusion:**

Physical activity is associated with lower social anxiety among rural-origin university students through both direct and indirect pathways. Body image satisfaction and social capital serve as critical statistical mediators–independently and sequentially. These findings support integrated, context-sensitive interventions addressing psychosocial determinants beyond physical activity alone.

## 1 Introduction

University life constitutes a pivotal developmental stage for young adults, characterized by academic pressures, challenges in social adaptation, and the ongoing process of self-identity formation. For students from rural backgrounds, this transitional period is especially intricate, giving rise to what scholars have conceptualized as a compounded “dual vulnerability” (Yu, 2022). Beyond the universal adjustment difficulties experienced by all undergraduates, these students encounter distinctive psychosocial stressors that stem from entrenched structural disparities between urban and rural contexts. With respect to cultural capital, nuanced differences–such as those in pedagogical norms, interpersonal etiquette, and aesthetic sensibilities–between students’ rural upbringing and the dominant university culture can engender experiences of cultural alienation and heightened anxiety (Li and Sun, 2023). Regarding socioeconomic capital, constrained familial resources often limit students’ ability to participate in extracurricular, social, and cultural activities that entail financial investment, thereby exacerbating their “sense of relative deprivation” (Zhang et al., 2022). In terms of social support networks, the primary systems traditionally anchored in local community and kinship ties frequently attenuate or disintegrate upon university entry; meanwhile, the development of new, secondary support networks is hindered by both limited relational competencies and restricted access to supportive opportunities (Liu and Shi, 2023).

The convergence of these factors gives rise to a distinctive pattern of psychological vulnerability, with social anxiety emerging as a particularly salient manifestation. Social anxiety is defined by persistent fear and avoidance of social situations, typically expressed through heightened nervousness, discomfort, and apprehension about potential negative evaluation in interpersonal interactions. It is often accompanied by somatic symptoms–such as increased heart rate, sweating, or gastrointestinal distress–that can impair academic engagement, hinder the development of meaningful relationships, and undermine overall psychological well-being (Zha et al., 2023; Zhang et al., 2025). Empirical evidence indicates that university students from rural backgrounds exhibit significantly higher levels of social anxiety relative to their urban peers–a disparity rooted in the dynamic interplay between macro-level structural inequities (e.g., educational resource disparities, geographic mobility constraints) and micro-level psychological processes (Li and Sun, 2023). Accordingly, identifying contextually appropriate interventions to mitigate social anxiety–interventions that are sensitive to rural-origin students’ unique socioeconomic constraints, cultural backgrounds, and psychological profiles–carries both substantial theoretical import and pressing practical relevance. Nevertheless, the precise psychological and social mechanisms underlying the association between physical activity and reduced social anxiety among rural-origin university students remain inadequately understood. In particular, the mediating roles of body image satisfaction and social capital–and, crucially, their sequential interrelationship–have yet to be empirically examined in this population. While existing domestic research has predominantly investigated isolated mediators in isolation, no study to date has tested or validated a serial mediation pathway wherein physical activity fosters body image satisfaction, which in turn enhances social capital, thereby attenuating social anxiety.

The relationship between physical activity and social anxiety has been extensively investigated in international research contexts. Studies conducted among Western populations have consistently demonstrated inverse associations between exercise engagement and social anxiety symptoms (Kandola et al., 2018; Martín-Rodríguez et al., 2024). Beyond cross-sectional evidence, longitudinal studies have further substantiated this relationship. A four-wave longitudinal study among 1,259 Chinese adolescents demonstrated that physical exercise significantly and negatively predicted subsequent social anxiety (Wu et al., 2025). Similarly, a three-wave cross-lagged study with 12-months follow-up found that physical activity and social anxiety exhibit bidirectional longitudinal associations: lower physical activity exacerbates social anxiety, whereas higher social anxiety reduces subsequent physical activity participation (Zhou and Yang, 2026). A systematic review and three meta-analyses further confirmed that longitudinal studies reported significantly lower social anxiety symptoms following physical activity interventions (Zika and Becker, 2021). Critically, a recent four-wave longitudinal study employing random intercept cross-lagged panel models demonstrated that physical exercise alleviates social anxiety through the mediating pathway of body image improvement, which directly supports the sequential associative framework examined in the present study (Xing et al., 2026). Participation in organized sports provides structured opportunities for social interaction and goal-directed behavior–factors that may mitigate social anxiety, as empirical evidence links sports involvement with enhanced psychological well-being (Wilzer et al., 2024). From a sociocultural perspective, social anxiety can be conceptualized as a maladaptive response to stressors related to peer acceptance and perceived social status (Haugan, 2023) –a framework especially salient for university students adapting to novel and complex social environments. Moreover, cross-national research indicates that social anxiety–recognized as a core feature of internalizing disorders–has shown a marked upward trend among adolescents and young adults. Emerging risk factors, including intensive digital media use and pervasive social technology engagement, are increasingly implicated in this rise (Keyes and Platt, 2024).

However, extant international research has seldom examined the distinct vulnerabilities faced by rural-origin students–a demographic of particular significance in rapidly urbanizing societies such as China. To date, scholarship has predominantly centered on urban populations in Western high-income countries, with comparatively little attention devoted to rural migrant groups navigating the complexities of rapid urbanization in low- and middle-income contexts. Moreover, the theoretical integration of the Cognitive-Behavioral Model of Body Image with Social Resource Theory–specifically to elucidate a sequential mediation pathway–remains critically underexplored in the global literature. The present study advances this body of work by: (a) investigating a non-Western setting characterized by culturally distinctive dynamics surrounding body image and social capital; (b) focusing explicitly on a vulnerable, understudied population–namely, university students of rural origin; and (c) empirically testing a novel sequential associative model that bridges psychological and sociological frameworks to capture the internal-to-external mechanism linking physical activity to reduced social anxiety.

As a proactive lifestyle choice, regular physical activity is robustly associated with wide-ranging benefits for both psychological and physiological well-being (Kandola et al., 2018; Martín-Rodríguez et al., 2024). These benefits extend beyond improvements in conventional physical health indicators to include reductions in negative affect, enhanced psychological resilience, and improved social adaptation (Lin et al., 2024; Liu et al., 2024). Among university students, participation in physical activity is increasingly recognized as an effective strategy for mitigating academic stress and navigating interpersonal challenges (Deng and Wang, 2024; Mu et al., 2024). Empirical evidence consistently demonstrates a significant negative association between physical exercise and social anxiety in student populations (Huang and Jiang, 2024; Jiang et al., 2025). This association is underpinned by dual mechanisms: direct physiological pathways–including neurochemical modulation and enhanced cerebral functional efficiency (Martín-Rodríguez et al., 2024) –and indirect psychological pathways mediated by factors such as increased psychological resilience (Li et al., 2024), heightened self-efficacy (Zhang et al., 2024), and improved emotion regulation capacity (Tang et al., 2022).

While existing research confirms the positive influence of sports participation on reducing social anxiety, the specific mechanisms underlying this association–particularly among university students from rural backgrounds–remain insufficiently examined. Notably, the potential mediating pathway linking sports participation to reduced social anxiety via body image satisfaction and social capital has received limited empirical attention. For this population, elucidating such a chain-associative pathway holds substantial theoretical explanatory power and practical implications for targeted intervention design. Body image satisfaction refers to an individual’s positive evaluation of their physical appearance and functional bodily capabilities (Cash et al., 2002). Contemporary theoretical frameworks have extended this foundational conceptualization. The Tripartite Influence Model (Thompson et al., 1999) posits that body image perceptions are shaped by three primary sociocultural forces–peers, parents, and media–which influence body dissatisfaction through the mechanisms of appearance comparison and internalization of idealized body norms. Recent meta-analytic evidence has consistently demonstrated that internalization of body shape ideals is strongly linked to body dissatisfaction across diverse populations (Paterna et al., 2021). Furthermore, a growing body of research has highlighted the protective role of positive body image constructs–such as functionality appreciation, defined as respecting and honoring the body for what it is capable of doing–which are consistently associated with fewer body image problems and better mental health outcomes (Linardon et al., 2023). In this study, body image satisfaction is operationalized using the Body Image States Scale. Students from rural origins may face distinct sociocultural pressures related to body image, stemming from (1) divergent aesthetic norms between urban and rural contexts; (2) heightened difficulty in conforming to urban-dominated ideals of the “ideal body”; and (3) differential embodied experiences–including early engagement in manual labor and inequitable access to sports infrastructure and programming (Yang et al., 2023). Empirical evidence consistently indicates a robust negative association between body image satisfaction and social anxiety: lower body image satisfaction intensifies fears of negative social evaluation and increases tendencies toward social withdrawal and avoidance (Liao et al., 2023).

Although body image satisfaction and social anxiety both center on concerns about negative evaluation, they represent conceptually and empirically distinct psychological constructs. Body image satisfaction refers specifically to an individual’s evaluative appraisal of their physical appearance and bodily capabilities, reflecting a domain-specific self-perception rooted in the body (Cash et al., 2002). In contrast, social anxiety involves a broader, more generalized fear of negative evaluation across diverse interpersonal situations–encompassing concerns about perceived social incompetence, rejection, and embarrassment during social interactions (La Greca and Lopez, 1998). Empirically, prior research has consistently demonstrated that, although these constructs are modestly correlated, they are statistically separable in confirmatory and exploratory factor analyses and exhibit divergent patterns of associations with theoretically relevant correlates–i.e., distinct nomological networks (Liao et al., 2023; Wan et al., 2024). In the present study, body image satisfaction is conceptualized as a specific, affectively laden self-evaluative state that may influence social anxiety through both direct effects and indirect pathways (e.g., via mediating cognitive or behavioral mechanisms), rather than as a redundant or overlapping construct.

Social capital refers to the collective resources and benefits that individuals or groups can access through their social networks and interpersonal relationships (Li et al., 2025). Upon entering university, students from rural backgrounds often face a dual challenge: relatively low baseline levels of social capital and significant difficulties in effectively mobilizing the limited resources they do possess. Participation in sports–especially team-based activities–offers a structured, inclusive, and socially de-stigmatized context for sustained interaction. As such, it is widely recognized as an effective mechanism for cultivating bridging social capital (Xiong and Zhou, 2025). Unlike bonding or linking forms, bridging social capital is grounded in shared activities, common goals, and mutual interests rather than inherited familial status or accumulated cultural capital. For students of rural origin, this asset provides a viable and equitable pathway to transcend structural social barriers and to build new, supportive networks within the collegiate environment (Deng and Wang, 2024).

Critically, students from rural backgrounds confront distinct body image challenges that stem from structural inequities in physical capital and divergent aesthetic norms. The prevailing urban-centric beauty ideal–centered on slimness, sartorial sophistication, and polished physical presentation–often stands in tension with the embodied realities of rural-origin students. Many of these students engaged in manual labor during childhood and had limited access to sports infrastructure, fitness education, and health-promoting resources prior to entering university. This disjunction can intensify body dissatisfaction and foster perceptions of physical inadequacy, thereby amplifying social anxiety within the university setting. To date, this distinctive body image predicament has received scant attention in international scholarship, which has largely centered on Western, urban populations.

For students from rural backgrounds, social capital assumes exceptional significance. Gaining admission to university often entails a near-total disconnection from their pre-existing kinship- and community-based support networks in rural settings. Concurrently, deficits in cultural capital frequently impede their capacity to navigate urban social norms and cultivate new interpersonal relationships. Within this context, bridging social capital–defined as social ties forged through shared activities rather than ascribed status–emerges as a critical mechanism for social integration. Distinct from bonding social capital, which is rooted in familial or inherited affiliations, bridging social capital is inherently egalitarian and accessible, thereby providing rural-origin students with a viable pathway to mitigate identity-based social exclusion.

Physical activity occupies a distinctive role in this process by offering a rare de-identified social space. On the sports field, individual worth is grounded primarily in athletic competence, effort, and collaborative engagement–rather than socioeconomic status, cultural background, or physical appearance. This equitable environment mitigates social anxiety and identity threat, enabling rural students to cultivate self-confidence and forge meaningful peer relationships within a low-stakes context. Investigating how physical activity may contribute to improved mental health through these dual mechanisms–enhanced body image and the accumulation of bridging social capital–can yield valuable insights for supporting the psychological adaptation of this underserved population.

However, a stronger theoretical rationale is needed to justify this specific sequential ordering–namely, that physical activity first correlates with higher body image satisfaction (BIS), which in turn correlates with greater social capital accumulation, ultimately correlating with lower social anxiety. Drawing on hierarchical self-concept theory (Shavelson et al., 1976), we posit that body image satisfaction, as a core dimension of physical self-concept, reflects a more fundamental and internalized form of self-evaluation. According to this framework, physical self-concept serves as a foundational layer of global self-concept and exerts a proximal influence on individuals’ social behaviors and relational functioning. Such positive self-perception is likely to develop prior to–and may even enable–the enactment of outward-oriented social behaviors–such as initiating conversations, cultivating trust, and participating in group activities–that are essential for building and sustaining social capital. In essence, a favorable body image may function as a psychological prerequisite, fostering the confidence necessary to actively seek, initiate, and maintain meaningful social connections.

This reasoning is consistent with emerging evidence from the broader literature on body image and mental health. For instance, a recent systematic review identified self-esteem as a key mediator in the relationship between body image disturbance and depression (Wang et al., 2024), suggesting that body image–related constructs often serve as foundational psychological mechanisms that precede downstream psychological or social outcomes. Similarly, within the present framework, body image satisfaction is posited as the primary intermediate variable–potentially facilitating the secondary association with social capital. This sequential pathway not only aligns with developmental models of self-concept formation but also provides a more nuanced account of how internal psychological changes may temporally precede and enable external social benefits in the link between physical activity and reduced social anxiety.

Grounded in hierarchical self-concept theory and social resource theory, this exploratory study aims to examine potential associations between physical activity and social anxiety among university students from rural backgrounds. Specifically, we investigate the potential associative roles of body image satisfaction and social capital in these relationships. The following preliminary hypotheses are proposed:

1. Greater levels of physical activity will be associated with lower levels of social anxiety.
2. Greater levels of physical activity will be associated with higher levels of body image satisfaction.
3. Greater levels of physical activity will be associated with higher levels of social capital.
4. We propose an exploratory sequential associative pathway for investigation: physical activity is statistically associated with body image satisfaction, which in turn is statistically associated with social capital, ultimately associated with reduced social anxiety.

It is important to note that, as a cross-sectional study, this design can only identify statistical associations and cannot establish causality or temporal precedence.

## 2 Materials and methods

This study adopted a cross-sectional design to examine the associations between physical activity and social anxiety among university students from rural backgrounds. All procedures were conducted in accordance with the ethical principles outlined in the Declaration of Helsinki for medical research involving human participants, as well as all applicable national laws, regulations, and institutional ethical standards. The study protocol was approved by the Institutional Review Board of Kunming University of Science and Technology (Approval No. KMUST-MEC-2026-003).

To ensure methodological rigor, all research staff responsible for survey administration underwent comprehensive training prior to data collection. Consistent with established best practices for cross-sectional research, a structured questionnaire was developed, incorporating socio-demographic items and psychometrically validated measures of physical activity, body image satisfaction, social capital, and social anxiety. The questionnaire began with a detailed study overview outlining its purpose, scope, and procedural framework. Following the acquisition of informed consent, an electronic version of the questionnaire was distributed via WeChat for anonymous, self-administered completion. The instrument opened with a formal informed consent statement, explicitly describing the study’s objectives, affirming the confidentiality of all collected data, and emphasizing the voluntary nature of participation.

Data were collected from September to October 2025. The target population comprised current undergraduate students enrolled in Chinese higher education institutions who held rural household registration (hukou) prior to university admission. A total of 1,102 questionnaires were returned. After excluding incomplete or invalid responses–defined as those completed in less than 300 s, exhibiting systematic response bias (e.g., straight-lining), or containing uniform answers across multiple items–the final analytic sample consisted of 965 participants, yielding an effective response rate of 87.6%. Missing data were handled using listwise deletion, given that the overall missingness rate was below 5%.

### 2.1 Participants and process

A convenience sampling strategy was employed to recruit participants. The questionnaire link was disseminated via social media platforms–primarily WeChat–to university students residing in Yunnan Province, China. Yunnan Province was selected for three principal reasons: (1) as a western Chinese province, it exhibits pronounced urban–rural disparities; (2) it hosts a substantial population of university students originating from rural areas; and (3) its distinctive multi-ethnic cultural landscape and high-altitude geographical setting give rise to unique patterns of sports participation and culturally embedded body image norms. To improve sample diversity, the survey link was disseminated via social media platforms–primarily WeChat–to university students residing in Yunnan Province, China. The questionnaire was circulated through student social networks, class group chats, and campus-related online communities, reaching students from four universities across three geographically distinct regions of Yunnan Province: Kunming University of Science and Technology (Kunming), Yunnan Normal University (Kunming), Lijiang University (Lijiang, northwest Yunnan), and Pu’er University (Pu’er, south Yunnan). These institutions were selected to cover a range of disciplinary profiles and geographical settings, including provincial capital and prefecture-level city contexts. Consistent with the convenience sampling strategy, the survey relied on voluntary participation through online dissemination channels without formal institutional coordination. It should be noted, however, that the use of convenience sampling may constrain the generalizability of the findings to the broader population of rural-origin university students across China.

The required sample size was determined through *a priori* power analysis for multiple regression models. A *post hoc* power analysis was subsequently conducted using the observed effect size from the final regression model–namely, R^2^ = 0.20 (f^2^ = 0.25)–which included four predictors: physical activity, gender, academic year, and left-behind experience. With a total valid sample of *N* = 965, α = 0.05, and target statistical power set at 0.80, the achieved power was >0.99. This indicates that the sample size was not only sufficient but substantially exceeded the minimum requirement to detect the observed effects with high confidence. According to Cohen’s (1988) conventions, f^2^ = 0.02, 0.15, and 0.35 correspond to small, medium, and large effect sizes, respectively; thus, the observed f^2^ = 0.25 reflects a medium-to-large effect. Moreover, the sample size satisfies–and far exceeds–the widely cited recommendation of 10–20 participants per item for exploratory or confirmatory factor analysis (Nunnally and Bernstein, 1994), given the 35-item structure of the questionnaire.

The demographic characteristics of the valid sample are summarized in Table 1. The sample comprised 531 male participants (55.0%) and 434 female participants (45.0%). By academic year, it included 300 first-year students (31.1%), 270 second-year students (28.0%), 231 third-year students (23.9%), and 164 final-year students (17.0%). Additionally, 512 participants (53.1%) reported having experienced a “left-behind” childhood or adolescence–defined as residing with grandparents or other non-parental relatives for more than six consecutive months while one or both parents worked long-term away from home (Hannum et al., 2018; Zhang et al., 2022).

**TABLE 1:** Demographic analysis results.

Variable	Category	Frequency	Percentage
Gender	Male	531	55.0%
	Female	434	45.0%
Grade	Freshman	300	31.1%
	Sophomore	270	28.0%
	Junior	231	23.9%
	Senior	164	17.0%
Left-behind experience	Yes	512	53.1%
	No	453	46.9%

### 2.2 Measures

All measures employed were well-established, widely validated scales. A standardized forward-translation and back-translation procedure was conducted to ensure cultural appropriateness and semantic equivalence of the Chinese versions. Except for demographic variables and physical activity, all constructs were assessed using Likert-type scales. This study measured only overall physical activity levels and did not differentiate among specific types (e.g., team versus individual sports; aerobic versus resistance training).

#### 2.2.1 Physical activity

The Physical Activity Rating Scale–3 (PARS-3), as revised by Liang (1994), was employed to assess physical activity levels. The scale consists of three items evaluating exercise intensity, duration, and frequency, each rated on a 5-point Likert-type scale. Intensity and frequency are scored from 1 to 5, whereas duration is scored from 0 to 4. A composite physical activity score is computed by multiplying the three item scores, yielding a total score ranging from 0 to 100. These scores were categorized descriptively as follows: low activity (0–19), moderate activity (20–42), and high activity (43–100). For regression analyses, the continuous raw PARS-3 score (0–100) was retained to maximize statistical power and preserve information–consistent with Liang’s (1994) recommendations for using the PARS-3 in regression-based modeling. The categorical classification was used exclusively for descriptive reporting.

#### 2.2.2 Body Image States

This scale–originally developed by Cash et al. (2002) and later revised and validated by Wei et al. (2017) –comprises six items. Participants rate their self-perceptions regarding appearance, physique, weight, bodily attractiveness, and feelings of self-worth relative to others on a 9-point Likert scale, ranging from 1 (extremely dissatisfied) to 9 (extremely satisfied). Items 2, 4, and 6 are reverse-scored; all relevant data were recoded accordingly prior to analysis. Higher total scores reflect greater body image satisfaction. In this study, we refer to this construct as body image satisfaction (BIS). The scale demonstrated excellent internal consistency in the present sample (Cronbach’s α = 0.915).

#### 2.2.3 Social capital

Drawing from the Social Capital Questionnaire developed by Chen (2022), which encompasses five dimensions–social network participation, volunteerism, trust, reciprocity, and norms–this study selected three dimensions aligned with Putnam’s conceptualization of bridging social capital: social network relationships, social trust, and norms. These three dimensions were chosen because they best capture the cross-group social connections and trust levels that university students can establish through shared physical activities, and are most closely aligned with the concept of bridging social capital that is central to this study. The original 24-item scale was reduced to 14 items following this selection. Responses were given on a 5-point Likert scale. The construct validity of this 14-item version was evaluated through confirmatory factor analysis (CFA), as reported in Section “3.2 Measurement model evaluation.” The CFA results demonstrated acceptable model fit, and all standardized factor loadings exceeded 0.70, supporting the convergent validity of the adapted scale. The scale exhibited very high reliability in this study (Cronbach’s α = 0.970). It should be noted that this adaptation provides only preliminary evidence of validity. Although CFA results supported acceptable model fit, the truncation of the original 24-item scale may introduce psychometric drift. Future research should complement CFA with exploratory factor analysis (EFA) and assess test–retest reliability across appropriate time intervals to further establish the psychometric properties and measurement invariance of this abbreviated scale.

#### 2.2.4 Social anxiety

The Social Anxiety Scale for Adolescents (SAS-A), originally developed by La Greca and Lopez (1998), consists of 18 items organized across three subscales and is rated on a 5-point Likert scale, yielding a total score ranging from 18 to 90. This study, however, employs a shortened version of the SAS-A, developed and validated by Sun et al. (2017). This 12-item version retains the original three-factor structure: (1) fear of negative evaluation, (2) social avoidance and distress in novel situations, and (3) social avoidance and distress in general situations. Responses are likewise scored on a 5-point Likert scale, resulting in a total score range of 12–60; higher scores indicate greater severity of social anxiety. In their validation study, Sun et al. (2017) reported strong psychometric properties for this abbreviated version: Cronbach’s α for the full scale was 0.874, and confirmatory factor analysis supported adequate model fit (CFI = 0.939; RMSEA = 0.076). In the present study, internal consistency was excellent, with Cronbach’s α = 0.964.

### 2.3 Statistical analysis

A standardized analytical procedure was adopted in this study to ensure the robustness and replicability of the findings. The initial data preprocessing phase involved a systematic process of screening and cleaning the collected data to address missing values, outliers, and inconsistencies. All statistical analyses were conducted using SPSS 26.0 and AMOS 24.0.

Descriptive statistics and bivariate correlation analyses were performed first; specifically, means, standard deviations, and Pearson correlation coefficients were computed for all study variables (see Table 2). Subsequently, confirmatory factor analysis (CFA) was conducted in AMOS 24.0 to evaluate the measurement model–comprising constructs of body image satisfaction, social capital, and social anxiety–with emphasis on assessing discriminant validity, factor loadings, and overall model fit indices.

**TABLE 2:** Descriptive statistics and correlation matrix for main variables (*N* = 965).

Variable	Mean	SD	PA	BIS	SC	SA
1. PA	37.17	24.85	–	–	–	–
2. BIS	5.53	1.62	0.502**			
3. SC	3.55	1.02	0.501**	0.523**		
4. SA	3.68	0.94	−0.443**	−0.396**	−0.449**	

PA, physical activity; BIS, body image satisfaction; SC, social capital; SA, social anxiety. **p < 0.01. All reliability coefficients are reported in Section “2.2 Measures.”

To examine potential sequential associative pathways among the variables, we employed the SPSS PROCESS macro (Model 6; Hayes, 2018), using the bias-corrected bootstrap method with 5,000 resamples. This approach enabled estimation of the total indirect effect, each specific indirect effect–including the sequential (i.e., multiple-step) indirect pathway–and their corresponding 95% bias-corrected confidence intervals. Statistical significance of any indirect effect was determined by whether its 95% confidence interval excluded zero. Given the cross-sectional design, all “mediation” analyses reported herein refer strictly to statistical associations and should not be interpreted as causal mechanisms or temporal sequences. Finally, to assess whether social anxiety levels differed significantly between students with and without left-behind experience, an independent-samples *t*-test was conducted.

Gender, academic year, and left-behind experience were included as control variables in all regression models, as prior research has consistently identified these factors as correlates of social anxiety among university students. All continuous predictors were standardized prior to model estimation to reduce the risk of multicollinearity and to enable direct comparison of effect sizes across variables. Multicollinearity was assessed using variance inflation factors (VIFs); all VIF values fell below 3, indicating no evidence of substantial multicollinearity. This study adheres to the STROBE (Strengthening the Reporting of Observational Studies in Epidemiology) statement for cross-sectional studies.

## 3 Results

### 3.1 Descriptive statistics and correlational analysis

Table 2 presents the means, standard deviations, and Pearson correlation coefficients for the primary variables. Reliability analysis revealed Cronbach’s α coefficients ranging from 0.915 to 0.970 across the three core construct scales, indicating excellent internal consistency of the measurement instruments. The sample demonstrated a moderate overall level of physical activity (*M* = 37.17, SD = 24.85). According to the official classification criteria of the PARS-3 scale, 27.9% of participants were classified as having low physical activity (0–19 points), 34.1% as moderate physical activity (20–42 points), and 38.0% as high physical activity (43–100 points).

Harman’s single-factor test was conducted to assess potential common method bias. An unrotated exploratory factor analysis (EFA) was performed on all 35 items comprising the four scales (PA, BIS, SC, SA). The results indicated that the first unrotated factor accounted for 47.57% of the total variance, which falls below the standard 50% threshold for indicating substantial common method bias (Podsakoff et al., 2003; Eichhorn, 2014). Nonetheless, the relatively elevated proportion underscores the prudence of including background covariates to insulate the model from potential inflation. Moreover, the inclusion of relevant demographic and experiential control variables in subsequent regression models further reduces the likelihood of systematic bias arising from common method variance. Collectively, these findings suggest that common method bias is unlikely to meaningfully compromise the validity of the observed relationships.

Regarding control variables, the sample comprised 531 males (55.0%) and 434 females (45.0%). Participants were distributed across academic years as follows: 300 first-year students (31.1%), 270 second-year students (28.0%), 231 third-year students (23.9%), and 164 fourth-year students (17.0%). With respect to left-behind experience, 512 participants (53.1%) reported having experienced parental absence during childhood or adolescence. All aforementioned demographic and background variables were included as covariates in the regression analyses to isolate the unique associations between physical activity and the outcome variables.

As shown in Table 2, the correlation analysis yields preliminary support for the study’s hypotheses. Significant associations emerged among the key variables in the theoretically predicted directions. Specifically, physical activity exhibited a strong positive correlation with body image satisfaction (*r* = 0.502, *p* < 0.01) and with social capital (*r* = 0.501, *p* < 0.01), and a moderate negative correlation with social anxiety (*r* = −0.443, *p* < 0.01). Correlation coefficients of approximately 0.50 suggest robust positive relationships between physical activity and both intermediate variables–indicating that higher levels of physical activity are meaningfully associated with more favorable body image perceptions and greater access to social resources. These findings imply that university students who engage more frequently in physical activity tend to report higher body image satisfaction, possess greater social capital, and experience lower levels of social anxiety. Furthermore, the two intermediate variables–body image satisfaction and social capital–were significantly and positively intercorrelated (*r* = 0.523, *p* < 0.01), and both demonstrated significant negative correlations with social anxiety (*r* = −0.396 and *r* = −0.449, respectively; both ps < 0.01), thereby satisfying the necessary conditions for subsequent regression analyses.

### 3.2 Measurement model evaluation

Prior to hypothesis testing, a confirmatory factor analysis (CFA) was conducted on the measurement model–comprising latent constructs of body image satisfaction, social capital, and social anxiety–using AMOS 24.0 to assess discriminant validity. Model fit indices were as follows: χ^2^ = 3355.180, df = 461, CFI = 0.906, RMSEA = 0.081 (90% CI [0.078, 0.083]), SRMR = 0.048. Although the RMSEA value marginally exceeds the stringent cutoff of 0.06 proposed by Hu and Bentler (1999), it falls within the widely accepted threshold of <0.08 recommended by Browne and Cudeck (1993), suggesting an adequate overall model fit. Moreover, both the CFI (≥0.90) and SRMR (<0.08) meet conventional criteria for acceptable fit, further supporting the adequacy of the measurement model. All standardized factor loadings of observed indicators on their respective latent variables exceeded 0.70, indicating strong convergent validity and an acceptable level of model fit.

In addition to model fit indices, we assessed composite reliability (CR) and average variance extracted (AVE) for each latent construct. CR values for body image satisfaction (BIS), social capital (SC), and social anxiety (SA) were 0.934, 0.973, and 0.969, respectively–each well above the conventional threshold of 0.70 (Fornell and Larcker, 1981). Similarly, AVE values were 0.704 (BIS), 0.723 (SC), and 0.721 (SA), all exceeding the recommended minimum of 0.50. Discriminant validity was further established using the Fornell–Larcker criterion: the square root of each AVE exceeded its corresponding inter-construct correlation with every other latent variable. Collectively, these findings provide robust support for both convergent and discriminant validity of the measurement model.

### 3.3 Sequential associative pattern analysis

Note: The following analyses report only statistical associations among variables. Due to the cross-sectional study design, causal inferences, temporal ordering, and statistical mediation (associative indirect effects) cannot be established as causal mechanisms.

To examine potential sequential associative patterns among physical activity, body image satisfaction, social capital, and social anxiety, regression analyses were conducted using the SPSS PROCESS macro (Model 6) with 5,000 bootstrap resamples. Gender, academic year, and left-behind experience were included as covariates.

The R^2^ values for the endogenous variables were as follows: body image satisfaction (R^2^ = 0.253), social capital (R^2^ = 0.354), and social anxiety (R^2^ = 0.282). In the total effect model–i.e., the model excluding mediators–physical activity and the covariates collectively accounted for 20.0% of the variance in social anxiety (R^2^ = 0.200).

The overall association between physical activity and social anxiety was statistically significant (unstandardized coefficient = −0.016 for the item-average score, 95% CI [−0.019, −0.014]; unstandardized coefficient = −0.192 for the total score, 95% CI [−0.228, −0.168]). However, the effect size was small: a 10-unit increase in physical activity (on a 0–100 scale) corresponded to only a 0.16-point reduction in the item-average social anxiety score (on a 1–5 scale) or a 1.92-point reduction in the total social anxiety score (on a 12–60 scale). Although statistically robust, this association demonstrates limited practical or clinical relevance in isolation.

**FIGURE 1:**
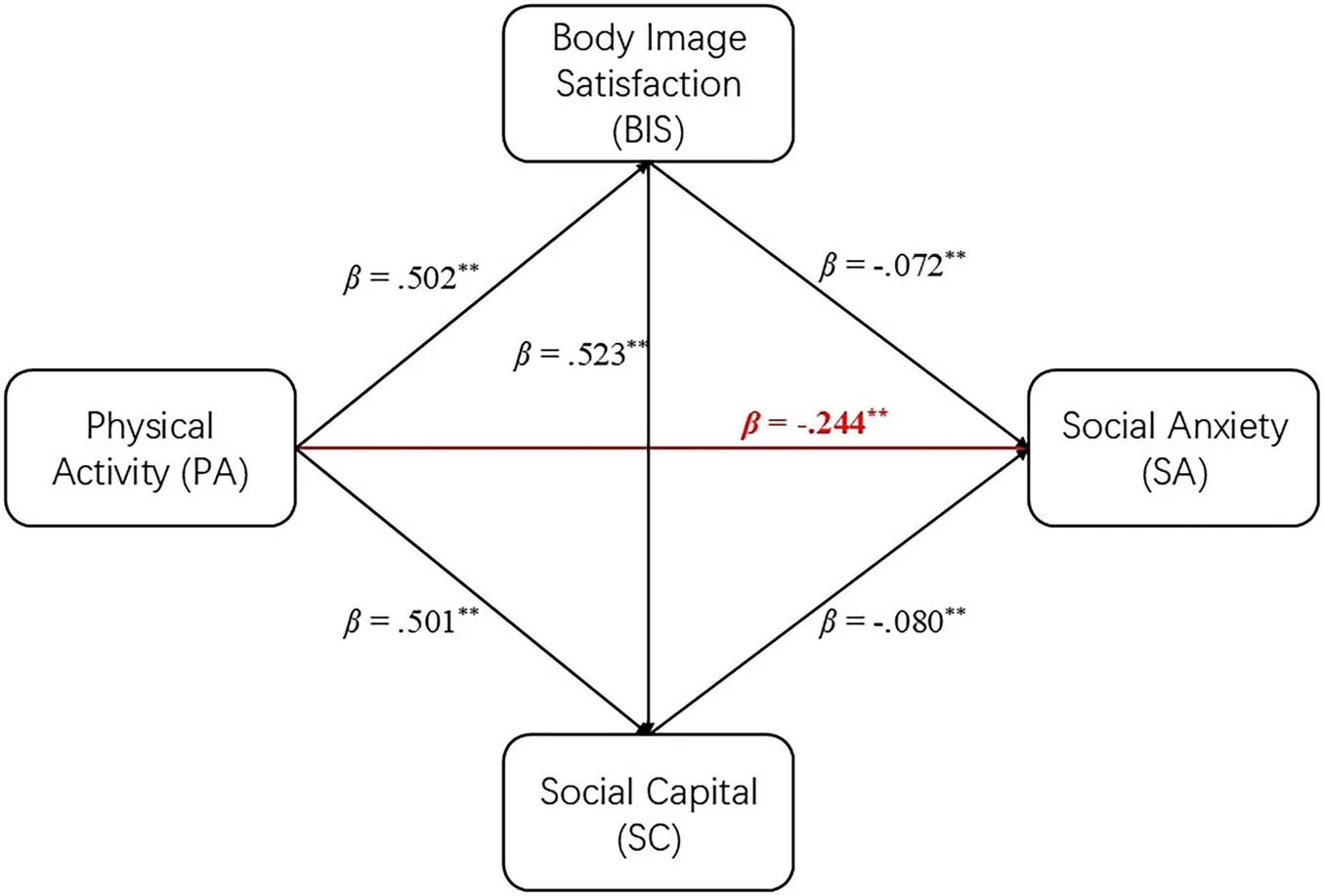
Path diagram of the sequential associative model with standardized coefficients (β). PA, physical activity; BIS, body image satisfaction; SC, social capital; SA, social anxiety. The model included gender, academic year, and left-behind experience as covariates (not shown for clarity). **p < 0.01. The direct effect (PA → SA) is shown in bold red. The sequential indirect effect (PA → BIS → SC → SA): β = −0.046 (95% CI [−0.028, −0.014]), accounting for 10.5% of the total effect.

After controlling for the intermediate variables, the direct association remained statistically significant (unstandardized coefficient = −0.009 for the item-average score, 95% CI [−0.012, −0.007]; unstandardized coefficient = −0.111 for the total score, 95% CI [−0.140, −0.081]). Figure 1 presents the path diagram of the sequential associative model with standardized coefficients for all pathways. Three distinct indirect pathways were identified, all yielding statistically significant bootstrap confidence intervals that excluded zero (see Table 3):

**TABLE 3:** Bootstrap analysis results of sequential associative patterns.

Path	Unstandardized effect	Standardized β	BootSE	BootLLCI	BootULCI
Total indirect effect	−0.090	−0.198	0.009	−0.108	−0.072
PA → BIS → SA	−0.033	−0.072	0.008	−0.049	−0.017
PA → SC → SA	−0.036	−0.080	0.006	−0.049	−0.025
PA → BIS → SC → SA	−0.021	−0.046	0.004	−0.028	−0.014
PA → SA (direct effect)	−0.111	−0.244	0.015	−0.140	−0.081
Total effect	−0.200	−0.443	0.013	−0.226	−0.175

PA, physical activity; BIS, body image satisfaction; SC, social capital; SA, social anxiety. Bootstrap samples = 5,000. CI, confidence interval. Unstandardized effects are based on raw total scores of all scales; standardized β coefficients are completely standardized effects. For item average score coefficients (used in the main text), divide the unstandardized values by 12 (number of items in the condensed Social Anxiety Scale). All confidence intervals do not include zero.

1. Physical activity is associated with greater body image satisfaction, which in turn predicts lower social anxiety.
2. Physical activity is associated with higher social capital, which in turn predicts lower social anxiety.
3. Physical activity is associated with greater body image satisfaction, which fosters higher social capital, thereby contributing to lower social anxiety.

Collectively, these three indirect pathways accounted for 45.0% of the total association between physical activity (PA) and social anxiety (SA). A summary of each indirect pathway, its standardized coefficient (cross-referenced with Table 3), and its percentage contribution to the total effect is presented below. This decomposition indicates that body image satisfaction (as indexed by BIS) and social capital serve as important statistical mediators (associative intermediaries) in the relationship between physical activity and social anxiety. Notably, the two single-mediator pathways contributed equally to the total effect, whereas the sequential, dual-mediator pathway–though smaller in magnitude–remained statistically significant.

### 3.4 Analysis of differences in left-behind experience

As shown in Table 4, an independent-samples *t*-test revealed a statistically significant difference in social anxiety scores between students with and without left-behind experience, *t*(963) = 2.105, *p* = 0.018. However, the effect size was negligible (Cohen’s *d* = 0.14), suggesting that left-behind experience exerts no meaningful practical influence on social anxiety levels in this sample. By conventional benchmarks (Cohen, 1988), *d* = 0.2, 0.5, and 0.8 represent small, medium, and large effects, respectively. This attenuated effect may be attributable to the high prevalence of left-behind experience in our rural-origin sample (53.1%), which diminishes between-group variability and thereby reduces the substantive interpretability of the observed statistical difference. In contexts where left-behind experience is normative rather than exceptional, it may cease to operate as a distinct risk factor for social anxiety.

**TABLE 4:** Differences in social anxiety scores between students with and without left-behind experience.

Variable	Left-behind experience	*N*	Mean	SD	t	*P*
SA	Yes	512	3.74	0.89	2.105	0.018
	No	453	3.61	0.98		

## 4 Discussion

### 4.1 Summary of key findings

This exploratory study investigated potential associative patterns between physical activity and social anxiety among 965 university students of rural origin in Southwest China. Consistent with our *a priori* hypotheses, we observed a significant direct negative association between physical activity and social anxiety, as well as three statistically significant indirect pathways–each statistically mediated (in an associative sense) by body image satisfaction and/or social capital. Notably, a sequential associative pathway emerged: higher levels of physical activity were associated with greater body image satisfaction, which in turn was statistically associated with higher social capital, ultimately linked to lower levels of social anxiety. Collectively, these indirect pathways accounted for 45.0% of the total association, underscoring the substantial contribution of psychological and social mechanisms in explaining the physical activity–mental health link in this population. Although all associations reached statistical significance, effect sizes were modest, and–given the cross-sectional design–causal inferences cannot be warranted.

### 4.2 Theoretical implications

The observed associative patterns are broadly consistent with predictions derived from hierarchical self-concept theory and social resource theory. The emergent sequential pattern suggests that body image satisfaction–functioning as a more foundational element of self-concept–may temporally precede the accumulation of social capital. This finding converges with prior evidence indicating that body image–related constructs frequently serve as antecedent mechanisms, shaping subsequent psychological and social outcomes (Wang et al., 2024).

This study advances the international literature by extending these theoretical frameworks to a distinct and underrepresented population: university students of rural origin in China. Our findings also align with and extend recent systematic evidence identifying body image–related constructs as key mediators in the physical activity–mental health relationship (White et al., 2024). By situating body image satisfaction within a sequential associative pathway that includes social capital, the present study provides a more nuanced understanding of how internal psychological states may translate into external social resources, thereby enriching both the Tripartite Influence Model and hierarchical self-concept theory. As summarized in Table 5, the two single-mediator pathways contributed equally to the total effect, while the sequential dual-mediator pathway-though smaller in magnitude-remained statistically significant. To our knowledge, it is the first empirical investigation to identify the sequential statistical mediating roles (associative pathways) of body image satisfaction and social capital in the association between physical activity and social anxiety within a non-Western cultural context. By integrating cognitive–behavioral perspectives on body image with sociological theories of social capital, the study offers a more integrative conceptual framework for understanding the mental health benefits of physical activity. Collectively, these findings highlight the necessity of accounting for structural inequities and cultural specificity when examining the physical activity–mental health nexus, as the underlying mechanisms may differ meaningfully across populations and sociocultural contexts.

**TABLE 5:** Summary of indirect pathways, standardized coefficients, and percentage contribution to total effect.

Pathway	Standardized β (from Table 3)	Percentage of total effect
PA → BIS → SA	−0.072	16.5%
PA → SC → SA	−0.080	18.0%
PA → BIS → SC → SA	−0.046	10.5%
Total indirect effect	−0.198	45.0%
Direct effect: PA → SA	−0.244	55.5%

PA, physical activity; BIS, body image satisfaction; SC, social capital; SA, social anxiety. Standardized coefficients (β) are reproduced from Table 3 for direct cross-reference. Percentages are calculated as the proportion of each indirect effect relative to the total effect. Due to rounding, percentages may not sum exactly to 100%.

### 4.3 Contextualizing the findings within rural-origin students’ adaptation

While the modest effect size of the overall association between physical activity and social anxiety constrains its utility as a standalone intervention, the implications of these findings gain greater salience when situated within the distinct challenges confronting university students from rural backgrounds.

First, for this population–characterized by multiple, intersecting vulnerabilities, including acculturative stress, socioeconomic disadvantage, and social isolation–even small protective factors may yield meaningful cumulative benefits over time. Social anxiety in this group arises from a complex confluence of adverse contextual, structural, and psychosocial determinants; thus, interventions targeting a single modifiable factor are unlikely to produce large-scale effects. Physical activity, however, stands out as a low-cost, widely accessible, and non-stigmatizing behavioral resource–making it one of the few empirically grounded, feasible mental health promotion strategies available to both resource-constrained institutions and students.

Second, physical activity is distinctive in its capacity to concurrently mitigate two salient barriers to psychological adaptation among rural students: negative body image and constrained social capital. In contrast to interventions that target only a single dimension, physical activity functions as an integrative platform–enhancing body satisfaction through the development of physical competence while simultaneously fostering bridging social capital via shared team-based experiences. This dual-mechanism efficacy renders physical activity a particularly valuable and efficient component of comprehensive, multi-tiered mental health support systems.

Finally, the relatively de-identified nature of sports spaces helps overcome a critical obstacle to social integration for rural students. In contexts where disparities in cultural capital frequently engender social stratification, athletic settings–such as basketball courts or soccer fields–constitute rare institutional spaces wherein status is conferred on the basis of effort, skill, and contribution rather than socioeconomic background or familiarity with dominant urban cultural norms. For instance, within a team sport like basketball, a student’s athletic performance and collaborative engagement are prioritized over their socioeconomic origins or cultural fluency. This meritocratic dynamic enables rural students to authentically demonstrate their competencies, earn peer recognition, and cultivate self-efficacy–thereby attenuating identity-based anxiety and strengthening pathways to social inclusion. Consequently, physical activity represents a uniquely promising vehicle for advancing both psychosocial well-being and equitable social participation among rural youth.

### 4.4 Practical implications

The findings of this study should be interpreted as hypothesis-generating rather than as evidence-based practice guidelines. Given the cross-sectional design, no causal inferences can be drawn, and the small observed effect sizes suggest that physical activity alone is unlikely to yield clinically meaningful reductions in social anxiety symptoms.

That said, the identified associative patterns warrant further investigation and suggest that future intervention research may benefit from evaluating comprehensive, multicomponent programs–integrating physical activity with targeted interventions addressing body image concerns and social skills development. Such integrated approaches may confer greater benefits for rural-origin university students than physical activity interventions delivered in isolation.

Based on our findings, we propose a three-tiered intervention framework for universities:

(1) At the institutional level, increase investment in accessible, inclusive sports facilities and organize regular inter-class and inter-departmental sports competitions to broaden opportunities for student engagement;
(2) At the community level, actively support the formation and sustainability of student-led sports clubs and societies–particularly those emphasizing cooperative play, shared goals, and sustained social interaction;
(3) At the individual level, embed evidence-informed positive body image education (e.g., 15-min weekly facilitated discussion sessions) into required physical education curricula and deliver targeted, empirically supported social skills training for students exhibiting elevated levels of social anxiety.

### 4.5 Limitations and future research directions

This study has several important limitations that warrant careful consideration:

1. Study Design Limitation: The cross-sectional design precludes causal inference and does not permit definitive conclusions regarding temporal precedence. Observed associations may reflect reverse causality (e.g., students with lower baseline social anxiety may be more likely to engage in physical activity) or confounding by unmeasured third variables. To clarify the direction and developmental trajectory of these relationships, future research should adopt longitudinal or experimental designs. Recent longitudinal evidence has begun to address this gap. Multi-wave longitudinal studies have demonstrated that physical activity negatively predicts subsequent social anxiety (Wu et al., 2025; Zhou and Yang, 2026), and that body image serves as a mediating mechanism in this relationship (Xing et al., 2026). These findings support the plausibility of our proposed sequential associative pathway and underscore the value of future longitudinal investigations to further validate the temporal ordering of the associations observed in the present study.
2. Effect Size Limitation: The overall association between physical activity and social anxiety is modest in magnitude, limiting its practical or clinical utility when considered in isolation. Similarly, the mean difference in social anxiety scores between students with and without left-behind experience is negligible. These small effect sizes underscore the need for cautious interpretation of the findings and highlight the importance of integrating contextual, individual, and environmental factors when designing future interventions.
3. Sample and Contextual Limitations: This study employed convenience sampling and recruited exclusively rural-origin students enrolled in universities across Yunnan Province, China. The province’s distinctive sociocultural and geographic context–including its multi-ethnic composition, high-altitude plateau terrain, and rich tradition of ethnic minority sports and outdoor physical activities–may have shaped the observed associations. For instance, the collectivist orientation inherent in many ethnic sports may foster greater social capital accumulation compared with the more individualistic forms of physical activity prevalent in other regions. Consequently, the findings may not be generalizable to urban populations, students from other provinces in China, or settings with divergent cultural, geographic, or institutional characteristics. Future research should adopt a multi-center design spanning diverse regions of China to systematically examine how local sports cultures, demographic profiles, and socioeconomic contexts moderate the relationships under investigation. Beyond the sociocultural specificity of Yunnan Province, recent cross-territorial research conducted across the geopolitically and ecologically diverse regions of Jammu, Kashmir, and Ladakh (*N* = 1,377) has demonstrated that geographical shifts significantly impact baseline physical activity, nutrition, and stress management behaviors among university cohorts (Wani et al., 2025a). This comparative evidence underscores the necessity of multi-center designs that account for regional heterogeneity–including variations in altitude, climate, and ethnic sporting traditions–in future investigations of territory-specific sports psychology. Moreover, climatic and environmental factors may moderate the psychological benefits of physical activity among rural-origin students adapting to urban campuses. The Healthy Lifestyle Scale (HLS), recently developed and validated for populations residing in climatically extreme regions with icy winters and dry summers, provides a structured SEM framework for understanding how extreme weather patterns alter individual health-promoting profiles (Wani et al., 2025b). This climate-sensitive measurement approach offers a valuable reference for future studies examining how high-altitude plateau environments–such as Yunnan’s–may shape or disrupt the psychological benefits of routine physical activity among vulnerable student populations.
4. Measurement Limitations: The adapted 14-item social capital scale was validated solely via confirmatory factor analysis (CFA). To strengthen its psychometric foundation and address potential psychometric drift, future studies should complement CFA with exploratory factor analysis (EFA) and assess test–retest reliability across appropriate time intervals. This is particularly important given that the original 24-item scale was substantially modified to isolate bridging social capital, which may affect the factor structure and measurement invariance across populations. Moreover, all constructs were measured using self-report instruments, raising the possibility of common method bias. Although Harman’s single-factor test indicated that such bias was unlikely to substantially affect the results, triangulation through multiple informants (e.g., peers, coaches, family members) or objective behavioral indicators (e.g., participation records, observational assessments) would enhance measurement rigor. Finally, physical activity was assessed only as an aggregate construct and was not disaggregated by type (e.g., team versus individual sports; aerobic versus resistance training). Given evidence suggesting that different modalities of physical activity may exert distinct influences on body image satisfaction and social capital development, future work should explicitly differentiate activity types to clarify their unique and interactive contributions.
5. Generalizability Limitation: The findings are specific to university students of rural origin and therefore cannot be generalized to students from urban backgrounds or to other populations. Future research should directly compare the associations between physical activity and mental health across rural and urban student groups to elucidate potential differences in underlying mechanisms. Moreover, future studies could examine how individual-level factors–including gender, academic discipline, and ethnic background–moderate the observed relationships.

## 5 Conclusion

By developing and empirically testing a sequential associative model, this study offers an in-depth examination of the mechanisms linking physical activity to social anxiety among university students of rural origin. Drawing on survey data from a representative sample of 965 such students, the research yields the following key findings:

First, physical activity is significantly associated with lower levels of social anxiety in this population. Specifically, the analysis reveals a direct negative association between physical activity and social anxiety–accounting for 55.5% of the total effect–as well as a significant sequential indirect pathway statistically associated with (mediated in the associative sense) body image satisfaction and social capital, which collectively explain 45% of the total effect. These findings provide robust empirical support for integrating physical activity–based interventions into mental health promotion strategies targeting rural-origin university students.

Secondly, this study introduces an integrated “body image–social resources” framework. Specifically, physical activity is associated with higher body image satisfaction through the body image pathway (PA → BIS → SA), which accounts for 16.5% of the total effect. In turn, enhanced body image satisfaction is statistically associated with more positive social engagement, which–via the sequential integration pathway (PA → BIS → SC → SA) – correlates with greater bridging social capital, explaining 10.5% of the total effect. Finally, increased social support derived through the social resources pathway (PA → SC → SA), accounting for another 18.0% of the total effect, is associated with reduced social anxiety. This sequential model integrates cognitive–behavioral theories of body image with social capital theory, highlighting a dynamic, temporally ordered interplay between internal psychological states and external social environments in the context of physical activity and mental health outcomes. The proposed sequential associative model thus elucidates the developmental trajectory from intrapsychic processes to interpersonal and structural social resources, offering a novel theoretical lens for understanding how physical activity influences mental health.

Thirdly, these findings hold important implications for understanding and supporting the psychological adaptation of rural-origin university students the framework suggests that physical activity may be statistically associated with reduced body image concerns arising from urban–rural sociocultural disparities, while concurrently correlating with the cultivation of equitable, inclusive, and supportive peer networks. This provides universities with an initial theoretical basis for designing holistic, integrative interventions grounded in a “mind–body–community” perspective–particularly programmes that synergistically combine team-based physical activities, evidence-informed body image promotion workshops, and structured initiatives aimed at strengthening bridging and bonding social capital.

In conclusion, this study advances the theoretical understanding of the associations between physical activity and mental health, providing an initial scholarly contribution that may inform strategies to enhance psychological well-being and quality of life among university students from rural backgrounds. Future longitudinal studies and controlled intervention trials will be essential to further validate the proposed model, thereby establishing a robust scientific foundation for designing targeted, evidence-based mental health interventions tailored to this population.

## Data Availability

The raw data supporting the conclusions of this article will be made available by the authors, without undue reservation.
